# Association of Dietary Fiber and Measures of Physical Fitness with High-Sensitivity C-Reactive Protein

**DOI:** 10.3390/nu16060888

**Published:** 2024-03-19

**Authors:** Ming-Zhen Su, Suyeon Lee, Dayeon Shin

**Affiliations:** Department of Food and Nutrition, Inha University, 100 Inha-ro, Michuhol-gu, Incheon 22212, Republic of Korea; sumingzhen123@naver.com (M.-Z.S.); suyean812@naver.com (S.L.)

**Keywords:** dietary fiber, physical fitness, high-sensitivity C-reactive protein (hs-CRP), metabolic equivalent of task (MET)

## Abstract

Dietary fiber intake and physical fitness are independently associated with high-sensitivity C-reactive protein (hs-CRP) levels. Nevertheless, the association between dietary fiber intake, measures of physical fitness, and hs-CRP levels has not yet been fully evaluated. We investigated the influence of a combination of dietary fiber intake and measures of physical fitness, including hand grip strength, resistance training, and metabolic equivalents of tasks, on hs-CRP levels. Data collected from the Korea National Health and Nutrition Examination Survey (KNHANES) spanning 2015 to 2018 were used in this study. A total of 16,934 participants (7434 men and 9500 women aged ≥19 years) were included in this study. After adjusting for confounding factors (age, education, income, marital status, smoking status, drinking habits, total energy intake, and aerobic physical activity), we employed a multivariable logistic model to examine the association of dietary fiber intake and measures of physical fitness with hs-CRP levels. Among women, the odds of high hs-CRP levels were lower in those with the highest dietary fiber intake and superior grip strength compared to in women with the lowest dietary fiber intake and weaker grip strength (odds ratio [OR] = 0.40, 95% confidence interval [CI] = 0.24–0.68). The highest dietary fiber intake who participated in resistance exercise at least three times per week had a reduced odds of high hs-CRP levels compared with those with the lowest dietary fiber intake who did not engage in resistance exercise in both men and women (OR = 0.53, 95% CI = 0.32–0.89; OR = 0.40, 95% CI = 0.19–0.84, respectively). Our findings indicate that dietary fiber intake and high levels of physical fitness were associated with reduced odds of elevated hs-CRP levels.

## 1. Introduction

High-sensitivity C-reactive protein (hs-CRP), present in the body during acute-phase inflammatory reactions, plays a crucial role in the immune system. Moreover, it controls molecular cellular viscosity and directly oversees the oxidation of low-density lipoproteins [1]. CRP is employed as an inflammatory marker to detect cardiovascular diseases due to its close relationship with myocardial infarction, stroke, and other diseases [2]. Elevated hs-CRP levels have been linked to chronic diseases, including obesity and cardiovascular disease [3,4], and metabolic syndrome, which disrupts insulin signal transmission, leading to insulin resistance [5,6,7]. A 3 mg/L cutoff has been reported to help identify individuals requiring more aggressive cardiovascular risk management, and this cutoff value has been incorporated into clinical guidelines, including those of the American Heart Association, as a marker for assessing cardiovascular risk [8]. A study conducted in Cuba reported that individuals with metabolic syndrome had hs-CRP levels four-fold higher than those without metabolic syndrome [9].

The importance of dietary fibers has been confirmed in several studies. Dietary fiber intake has been reported to maintain the balance of microorganisms in the gut, promote the production of short-chain fatty acids (SCFA), reduce diarrhea and constipation, and enhance immunity [10,11,12,13,14,15]. In a study conducted in Portugal, serum hs-CRP concentration was significantly reduced by 30% for every 100 g of vegetables rich in dietary fiber consumed by normal-weight men [16]. Research involving Africans and Mexican Americans showed that individuals with the highest dietary fiber intake had a 41% lower likelihood of having a hs-CRP level > 3.0 mg/L compared to those with the lowest dietary fiber intake [17]. A cross-sectional study comprising Finnish adults determined that for every 50 g rise in whole-grain intake per day, hs-CRP concentration decreased by 0.12 mg/L, while for every 50 g increase in refined grain consumption, hs-CRP concentration increased by 0.23 mg/L [18].

An individual’s level of physical fitness has notable associations with inflammation. Hand grip strength, one of the physical fitness measures, serves as a major indicator of overall strength and effectively reflects the overall strength of the entire body [19,20]. In a cross-sectional study of Korean adults aged 50 years or older, hs-CRP levels were inversely associated with grip strength in men, with grip strength decreasing linearly across increasing serum hs-CRP quartiles [21]. Another study in hospitalized patients who are non-critically ill also found that CRP was an independent predictor of grip strength, with grip strength being significantly lower in patients with inflammation [22]. Resistance training is also positively associated with anti-inflammatory properties. A previous meta-analysis showed that resistance training’s anti-inflammatory effects and increased muscle mass tended to reduce levels of CRP, an inflammatory marker [23]. Long-term combined aerobic and resistance exercise reduced subclinical inflammatory markers in healthy young men, and the higher the frequency of this exercise, the more beneficial the anti-inflammatory effects of physical activity [24]. Furthermore, people with long-term exercise habits have significant anti-inflammatory properties compared to those without exercise habits, and several studies have shown a negative association between physical activity and hs-CRP [25,26,27,28,29,30,31,32]. A study comprising US men showed that increased participation in weekly swimming, running, and cycling was associated with a proportional decrease in their serum hs-CRP levels [33]. A study of 58-year-old Swedish men reported that moderate physical activity, such as walking or cycling at least 4 h a week, or intense physical activity, such as running and swimming weekly, was associated with lower hs-CRP levels compared to sitting for long periods of time, such as reading a book or watching TV [34]. Another random sample of Finnish adults found that self-rated physical fitness and estimated aerobic fitness were negatively associated with levels of systemic inflammation in both men and women, and furthermore, physical activity during commuting and leisure time was also negatively associated with CRP levels in women [35]. Therefore, elevating physical fitness levels may significantly contribute to a reduction in inflammation. 

Collectively, the existing literature illustrates the negative association between hs-CRP levels and both dietary fiber intake and physical fitness levels. However, the relationship between dietary fiber intake and measures of physical fitness and the ideal combination for reducing hs-CRP levels remain relatively unexplored. Therefore, this study aims to investigate the association between daily dietary fiber intake and various aspects of physical fitness measurement, including hand grip strength, resistance training, total physical activity levels, and hs-CRP levels.

## 2. Materials and Methods

### 2.1. Sources of Data and the Participants in This Study

**Figure 1:**
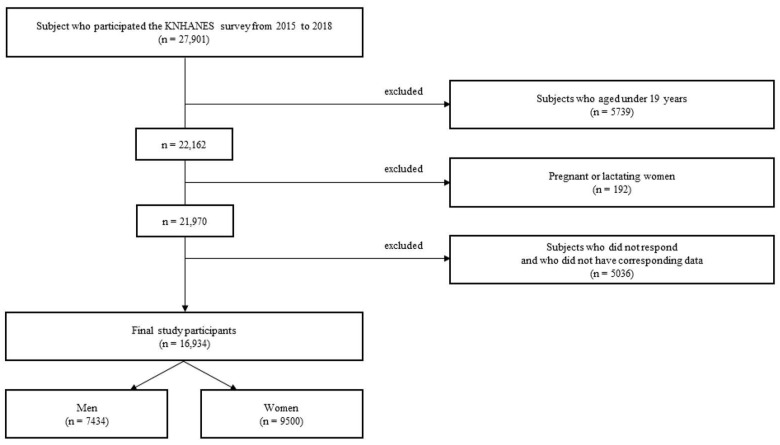
Flowchart of participant selection for this study.

The Korea National Health and Nutrition Examination Survey (KNHANES) is a population-based, cross-sectional survey to assess the health and nutritional status of the Korean population. At the mobile medical examination center, trained and qualified medical personnel conducted the KNHANES health interviews and physical tests. Each participant provided written informed consent before participating in the survey [36]. Of the 27,901 patients enrolled in the KNHANES between 2015 and 2018, those aged <19 years (n = 5739), pregnant or lactating women (n = 192) [37], and those who did not respond or did not have data (n = 5036) were excluded. A total of 16,934 participants (7434 men and 9500 women) were included in the final analysis (Figure 1).

### 2.2. Statement on Ethics and Availability of Data

The KNHANES was conducted following the principles outlined in the Declaration of Helsinki, and approval for this study was granted by the Research Ethics Review Committee of the Center for Disease Control and Prevention (2018-01-03-P-A).

### 2.3. Socio-Demographic and Lifestyle Factors

Socio-demographic data included age, education level, income, marital status, smoking status, and drinking habits, and aerobic physical activity status. This study was conducted on Korean adults aged ≥19 years. Four educational levels were considered: elementary, middle school, high school, and university or higher. Incomes were divided into low, middle-lower, middle-upper, and high. Marital status was categorized as married or unmarried. Smoking status was classified into three distinct groups: current (including both regular and occasional smokers), past (former smokers who were not currently smoking), and never smoked (individuals who had never smoked). As for drinking habits, individuals who had not consumed alcohol in the previous year and had no drink per month were classified as non-drinkers; those who had one, two, or three drinks per month were classified as moderate drinkers; and those who drank ≥2–3 times per week were considered high drinkers. Aerobic exercise status was marked as “yes” if individuals engaged in more than 2 h 30 min of moderate-level exercise, 1 h 15 min of high-level exercise per week, or a combination of both (where 1 min of high-level exercise is considered equivalent to 2 min of moderate-level exercise), and “no” if the specified criterion was not met.

### 2.4. Anthropometric Measurements

Waist circumference was measured using an instrument with a minimum of 0.1 cm and a maximum of 200 cm [38]. Waist circumference measurements were obtained after exhalation. Using a water-based pen, a mark was applied at two locations on the participant’s side, one at the lower end of the last rib segment and the other at the upper end of the long bone ridge. Waist circumference was subsequently measured at the midpoint between the designated points. A tape measure was placed horizontally on the ground, and the participant was instructed to speak to relieve tension. Abdominal obesity was defined as a waist circumference of ≥90 cm for men and ≥85 cm for women; the opposite was considered within the normal range [38,39]. Body mass index (BMI) was obtained by dividing weight in kilograms by the square of height (kg/m^2^). Categories of BMI were as follows: <18.5 kg/m^2^, underweight; >18.5 kg/m^2^ and <23 kg/m^2^, normal weight; ≥23 kg/m^2^ and <25 kg/m^2^, overweight; and ≥25 kg/m^2^, obese [40,41]. 

### 2.5. Assessment of Inflammation

Fasting blood from participants (fasted after 7:00 p.m. the day before the survey) was used for testing [42]. Blood samples were mixed in serum separator tubes approximately five times in reverse, placed vertically for 30 min to coagulate, and centrifuged at 3000 rpm for 15 min to obtain the serum from the top layer. Serum hs-CRP concentrations were quantified using an immunoturbidimetric method with a reference standard between 1.0 and 200.0 mg/L with a Cobas (Roche/Germany) instrument [43,44]. The inflammation level was classified based on hs-CRP levels, where <3 mg/L indicated a low inflammatory state and ≥3 mg/L indicated a high inflammatory state [45]. In this study, hs-CRP levels ≥3 mg/L were considered high.

### 2.6. Dietary Assessment

A single 24 h dietary recall was used to assess the dietary fiber intake (g/day) [46]. Based on this information, dietary fiber intake was divided into four groups: quartile 1 (0–14.71 g), quartile 2 (14.72–22.46 g), quartile 3 (22.47–32.12 g), and quartile 4 (32.13–228.04 g) in men; and quartile 1 (0.07–12.70 g), quartile 2 (12.71–19.01 g), quartile 3 (19.02–27.94 g), and quartile 4 (27.95–148.53 g) in women [47].

### 2.7. Assessment of Physical Fitness and Sedentary Time

The Global Physical Activity Questionnaire (GPAQ) is a reliable tool for evaluating a broad range of physical activities [48,49,50,51,52]. Our analysis included five areas of physical activity: high- and moderate-intensity work, transportation, and high- and moderate-intensity leisure. Moderate activity consumes approximately four fold as many calories as sedentary activity, whereas high activity consumes approximately eight fold as many calories as sedentary activity. To calculate an individual’s overall energy expenditure using GPAQ data, we assigned four and eight metabolic equivalents of tasks (MET) to moderate and vigorous exercises, respectively [53,54]. 

High-intensity work (MET) = 8.0 METs × days/week × minutes/day;

Moderate-intensity work (MET) = 4.0 METs × days/week × minutes/day;

High-intensity leisure (MET) = 8.0 METs × days/week × minutes/day;

Moderate-intensity leisure (MET) = 4.0 METs × days/week × minutes/day;

Transportation (MET) = 4.0 METs × days/week × minutes/day;

Total physical activity (MET) = high-intensity work + moderate-intensity work + high-intensity leisure + moderate-intensity leisure + transportation.

Based on the MET, we classified participants with total physical activity levels of 0–249 min per week as inactive, 250–499 min per week as somewhat active, 500–999 min per week as active, and ≥1000 min per week as very active [53,54,55]. Resistance training (the frequency of strength training sessions per week, primarily involving barbells, dumbbells, or push-ups) was categorized as none (0 times per week), ≥1 and <3 times per week, or ≥3 times per week. 

To test hand grip strength, the participants stood forward in a standing position and straightened their waists. Their shoulders were straight, their arms hung down naturally, their arms or wrists were not bent, and their arms were on their sides and did not touch the body. The legs and pelvis were wide apart, with the feet in front. Hand grip strength was measured three times in each hand using a digital grip dynamometer (TKK 5401; Takei Scientific Instruments Co., Ltd., Tokyo, Japan), and the final grip strength value was the average of three measurements for the left and right hands, respectively [56,57]. Koreans predominantly use their right hand [58], and grip strength measured on the dominant hand was defined as maximum grip strength [59]. Dominant hand grip strength in men was divided into low (5.27–32.83 kg), medium (32.86–40 kg), and high (40.03–69.70 kg). Dominant hand grip strength in women was divided into low (5.27–19.43 kg), medium (19.46–23.96 kg), and high (23.97–44.70 kg) [60]. Sedentary time was assessed based on participants’ self-reported sedentary time, which was divided into quartiles based on the number of hours spent sitting per day. 

Sedentary time (hours/day) was divided into quartiles for men and women, and the categories were as follows: 0–2 h, 3–6 h, 7–10 h, and 11–22 h for men and 0–3 h, 4–6 h, 7–10 h, and 11–20 h for women. 

### 2.8. Statistical Analysis

In this study, we employed various statistical analyses to explore the association among participant characteristics, inflammation status, and physical fitness levels. The *t*-test and chi-square test were used to explore the association between the overall traits and inflammation status. We employed one-way analysis of variance (ANOVA) and chi-square tests to assess the relationship between participant characteristics and levels of total physical activity. Additionally, we investigated the combined impact of dietary fiber intake and physical activity on hs-CRP (mg/L) levels. Multivariable logistic regression analysis was conducted to compute the adjusted odds ratios (OR) with 95% confidence intervals (CI). Two models were employed: Model 1 without adjustment and Model 2 adjusted for various factors such as education, income, age, marital status, drinking habits, smoking status, aerobic exercise practice rate, and total daily energy intake. Statistical Analysis System 9.4 (SAS, Cary, NC, USA) was used for statistical analyses, and significance levels were set at a *p*-value < 0.05.

## 3. Results

### 3.1. Characteristics of the Study Participants Based on hs-CRP Levels and the Category of Total Physical Activity

Table 1 describes the general characteristics of the participants categorized by sex and hs-CRP levels. For both men and women, participants with hs-CRP greater than 3 mg/L were older, more likely to have obesity and abdominal obesity, more likely to be married, and had lower levels of education (all, *p* < 0.0001). Women with a hs-CRP level of ≥3 mg/L exhibited higher total energy (kcal/day), carbohydrate, and fat (g/day) intake, along with lower protein and dietary fiber (g/day) intake compared to those in women with a hs-CRP level of <3 mg/L. Conversely, men with a hs-CRP level of ≥3 mg/L demonstrated lower total calorie (kcal/day), carbohydrate, protein, fat, and dietary fiber (g/day) intake compared to those in men with a hs-CRP level of <3 mg/L. Specifically, among men, the hs-CRP levels were lower in those who participated in aerobic exercise compared to those who did not.

Table 2 displays the general traits of the participants according to sex and total physical activity categories based on total physical activity level. In men, age, abdominal obesity status, hs-CRP, and intake of energy, carbohydrate, protein, fat, dietary fiber all significantly differed by the levels of total physical activity (all, *p* < 0.05). In women, age, abdominal obesity status, and intake of energy, carbohydrate, protein, fat, dietary fiber all significantly differed by the levels of total physical activity (all, *p* < 0.0001).

### 3.2. Association of Dietary Fiber Intake and Sedentary Time with Inflammation

Table 3 shows the incidence of elevated hs-CRP levels according to sedentary time and dietary fiber intake. Women in the highest quartile of dietary fiber intake had significantly lower odds of having high hs-CRP levels compared to women in the lowest quartile, specifically when the sedentary time ranged from 11 to 20 h (OR = 0.57, 95% CI = 0.33–0.99). This result was statistically significant after adjustments for age, income, education, drinking habits, smoking, marital status, aerobic exercise practice rate, and total daily energy intake (OR = 0.32, 95% CI = 0.16–0.63).

### 3.3. Association of Dietary Fiber Intake and Hand Grip Strength with Inflammation

Table 4 shows the incidence of elevated hs-CRP levels categorized according to dietary fiber intake and hand grip strength. In both men and women, individuals with highest dietary intake and highest hand grip strength were found to have significantly lower odds of elevated hs-CRP levels compared to individuals with the lowest dietary fiber intake and lowest hand grip strength (OR = 0.61, 95% CI = 0.40–0.94; OR = 0.43, 95% CI = 0.28–0.64, respectively). In men, the results were not statistically significant after adjusting for confounding variables. However, in women, the association remained statistically significant after adjustment for age, income, education, drinking habits, smoking, marital status, aerobic exercise practice rate, and total daily energy intake (OR = 0.40, 95% CI = 0.24–0.68).

### 3.4. Association of Dietary Fiber Intake and the Number of Days of Resistance Training per Week with Inflammation

Table 5 shows the odds ratio of high hs-CRP levels according to dietary fiber intake and engagement in resistance training. For both men and women, the odds of high levels of hs-CRP were significantly lower in participants with the highest quartile of dietary fiber intake who engaged in resistance training ≥3 times per week compared to in participants with the lowest quartile of dietary fiber intake without resistance training (OR = 0.56, 95% CI = 0.37–0.85; OR = 0.40, 95% CI = 0.21–0.77, respectively). The results retained significance even after accounting for confounding factors such as age, income, education, drinking habits, smoking, marital status, aerobic exercise practice rate, and total daily energy intake in both men and women (OR = 0.53, 95% CI = 0.32–0.89; OR = 0.40, 95% CI = 0.19–0.84, respectively).

### 3.5. Association of Dietary Fiber Intake and Total Physical Activity with Inflammation

Table 6 shows the odds ratio of high hs-CRP levels based on total physical activity levels and dietary fiber intake. Among inactive women, those in the highest quartile of dietary fiber intake had a significantly lower odds of high hs-CRP levels compared with those in the lowest quartile (OR = 0.66, 95% CI = 0.47–0.91). These findings remained significant even after accounting for confounding factors (OR = 0.49, 95% CI = 0.32–0.75).

## 4. Discussion

In this study, dietary fiber intake was inversely related to elevated hs-CRP levels, aligning with previous research [17,61,62,63,64,65]. In a cross-sectional analysis conducted in the US encompassing 524 individuals, a negative association was depicted between total dietary fiber intake and hs-CRP concentrations. Participants in the highest quartile of total fiber intake were observed to have a 63% lower likelihood of elevated CRP compared to individuals in the lowest quartile (OR = 0.37, 95% CI = 0.16–0.87) [46]. Although the mechanisms underlying the anti-inflammatory effects of dietary fibers are unclear, insights from a literature review suggest that high-fiber diets could reduce inflammation by altering intestinal pH and permeability [66]. Additionally, an animal experimentation mouse study suggested that dietary fiber could regulate the gut microbiome, which causes changes in the production of SCFAs, thereby reducing inflammation [67]. Another study conducted in the US evaluating the importance of dietary fiber observed that women were more sensitive to satiety in high-fiber diets than men, which has implications for regulating energy intake and preventing obesity [68]. This was confirmed by another cross-sectional study performed in Europe, revealing that the caloric intake during dinner following a high-fiber diet at lunch was 18% lower than when eating a low-fiber diet [69]. A cohort study conducted in France and Belgium showed that a 10% increase in BMI was associated with a 20% increase in hs-CRP levels [70]. Dietary fiber mainly reduces the occurrence of metabolic problems such as obesity by regulating the gut flora and improving satiety (resulting in reduced calorie intake), thereby reducing the prevalence of high inflammation [71].

To the best of our knowledge, this is the first study to show that dietary fiber intake and hand grip strength are related to hs-CRP levels. In our study, women with the highest dietary fiber intake and strongest hand grip strength reported lower odds of high hs-CRP levels compared to those with the lowest dietary fiber intake and weakest hand grip strength. Previous studies examined the association between hand grip strength and hs-CRP levels. An examination of Korean women indicated an independent and inverse association between hs-CRP levels and grip strength, and individuals with low grip strength demonstrated increased odds of elevated serum hs-CRP levels compared to those with high grip strength [72]. In a comprehensive meta-analysis, an investigation into the association between inflammatory markers and skeletal muscle strength revealed an inverse relationship between hs-CRP levels, hand grip strength, and knee extension strength [73]. Moreover, a Swiss study revealed a consistent inverse association between grip strength and hs-CRP levels. For every 5 kg increase in grip strength, hs-CRP levels decreased by 6.8% in women and 3.2% in men [20]. In a separate study conducted in Korea, it was observed that for every unit increase in absolute grip strength, hs-CRP levels decreased by 0.02 mg/dL. Additionally, their findings indicated a positive association between relative grip strength and overall health status [74]. In a cross-sectional study conducted in Colombia and Portugal, individuals with optimal adherence to the Mediterranean diet and high muscle status, as reflected by hand grip strength, exhibited the lowest average levels of hs-CRP [75]. Collectively, elevated grip strength is linked to lower hs-CRP levels, particularly when combined with high dietary fiber intake. 

Based on our results, men and women who had the highest dietary fiber intake and engaged in resistance training ≥3 times a week had lower odds of elevated hs-CRP levels. In a study encompassing Iranian women, 22 participants were assigned to either a control group or a resistance training group (three times a week for eight weeks). The resistance training group exhibited significantly lower hs-CRP levels compared to the control group [76]. Moreover, the results of a study from Brazil demonstrated a substantial decrease in hs-CRP (−0.45 ± 0.43 mg/L) in 48 individuals with obesity who were given a fiber-rich diet combined with physical activity (mainly cycling and weight training) at least three times a week [77]. Similarly, a study focusing on overweight adults in Brazil observed that a 10-week intervention involving a high-fiber dietary pattern (30 g of fiber per day) combined with resistance training three times a week led to a modest reduction in hs-CRP values (0.51 ± 0.53 mg/dL) compared to pre-intervention levels (0.54 ± 0.51 mg/dL) [78]. In summary, resistance training was negatively associated with hyperinflammation and may lower hs-CRP levels when combined with high-fiber diet, which is consistent with our study findings.

Our results demonstrate a clear association between increased dietary fiber intake and increased total physical activity, which is associated with a lower likelihood of elevated hs-CRP levels. A substantial body of literature consistently supports the association of increased dietary fiber intake and high physical activity levels with a diminished risk of severe inflammation [25,26,28,35,79]. Although both dietary fiber and physical activity reduce the risk of increased inflammation, their mechanisms of action differ. Dietary fiber primarily reduces inflammation by affecting digestion and absorption and regulating the health of the gut flora. Physical activity reduces inflammation through energy expenditure, improving muscle development, cardiovascular health, and metabolism. A longitudinal study conducted in Israel reported that aerobic exercise training reduced hs-CRP levels and independently suppressed low-grade inflammation throughout the body [80]. Therefore, physical activity can directly reduce inflammation by improving the health of the cardiovascular system. Physical activity has demonstrated anti-inflammatory effects by reducing the incidence of cardiovascular diseases associated with elevated hs-CRP levels [81]. A study conducted in the US demonstrated a 25% variance in hs-CRP levels among men and a 68% difference among women, thereby differentiating individuals with high levels of physical activity from those with low physical activity levels [82]. Another US study reported that combining a healthy diet (assessed using the healthy eating index) with physical activity resulted in a greater reduction in hs-CRP levels than either factor alone. Participants meeting both physical activity and healthy eating requirements showed lower hs-CRP levels (β = −0.34). The study revealed no synergy between a healthy diet and physical activity levels; each was independently associated with hs-CRP levels [83]. A cross-sectional study evaluating the combination of the Athens Mediterranean diet and moderate physical activity documented a 72% decrease in the prevalence of high hs-CRP levels [84]. Dietary fiber is an integral component of the Mediterranean diet, with a daily intake of 33 g [85,86]. A study of obese women in the US reported that individuals who followed a reduced-calorie dietary pattern and simultaneously performed 225 min of physical activity per week had lower hs-CRP by 0.87 mg/L (41.7%) decrease in hs-CRP compared to the control group [87]. In summary, physical activity combined with a healthy diet is strongly associated with reduced odds of severe inflammation.

Our findings also showed an association with hs-CRP not only from dietary fiber, but also from other nutrients such as carbohydrates, proteins, and fats. A combination of these other nutrients and physical activity may affect hs-CRP. A study in obese and overweight women reported that a high-protein diet combined with physical activity had a significant effect on reducing hs-CRP levels [88]. Also, one previous study found that a low-fat diet combined with physical activity including brisk walking, jogging, or running was associated with lower CRP levels in women with metabolic syndrome [89].

Although dietary fiber has been shown to reduce serum hs-CRP levels, there is a scarcity of studies examining its relationship with prolonged periods of sitting. In our study, women who sat the longest showed lower serum hs-CRP levels when they had the highest dietary fiber intake, as opposed to when they had the lowest dietary fiber intake. In the US, sitting for more than 4 h a day has been linked to increased BMI and obesity, contributing to elevated hs-CRP levels [90]. Another US cross-sectional study observed a 35–41% risk reduction in all-cause deaths and a 32–61% risk reduction in cardiovascular deaths when sitting for <6 h a day combined with high dietary fiber and vitamin intake [91]. A cross-sectional investigation involving 10 European countries indicated that following a Mediterranean diet might alleviate inflammation related to a sedentary lifestyle [92]. Consequently, there is a pressing need to evaluate the association between dietary fiber intake and serum hs-CRP concentrations in individuals who engage in extended sitting [93].

This study had several limitations. First, as a cross-sectional study, it can only establish an association between dietary fiber, physical activity, and hs-CRP. Detecting the change over time is challenging in this context, preventing the determination of a causal relationship. Second, our study used average hand grip strength values in the analysis, which did not take into account individual body weight. Third, only one inflammatory biomarker (hs-CRP) was used in this study, resulting in an incomplete understanding of inflammation. Fourth, we did not exclude a few individuals with underlying diseases. These conditions are prone to cause a substantial rise in hs-CRP levels, and individuals with underlying health conditions may likely exhibit deviations from normal levels of physical activity, which could have influenced the data. Nevertheless, our study has several strengths, including its robust and nationally representative sample size, encompassing the Korean population from 2015 to 2018. Furthermore, we segmented the level of grip strength, frequency of resistance training, and total physical activity levels in terms of physical fitness. Our findings provide insights into the association between physical fitness, dietary fiber intake, and hs-CRP levels, which may be of public interest.

## 5. Conclusions

In conclusion, our study found a significant association between increased dietary fiber intake and increased physical activity, which was associated with a lower likelihood of elevated hs-CRP levels. In particular, women who consumed the most dietary fiber and had the highest hand grip strength or who consumed the most dietary fiber and performed resistance training three or more times had 60% lower odds of high inflammation. To advance our understanding, future studies should elucidate the underlying mechanisms by which dietary fiber and physical activity influence inflammation levels and investigate potential sex-specific outcomes.

## Figures and Tables

**Table 1:** Characteristics of the study participants based on hs-CRP levels.

	Men				Women			
Variable	Total (n = 7434)	hs-CRP <3 mg/L (n = 6731)	hs-CRP ≥3 mg/L (n = 703)	*p*-Value ^a^	Total (n = 9500)	hs-CRP <3 mg/L (n = 8787)	hs-CRP ≥3 mg/L (n = 713)	*p*-Value ^a^
Age (years)	45.69 (±0.26)	45.35 (±0.74)	49.27 (±0.72)	<0.0001	47.66 (±0.25)	47.57 (±0.77)	48.85 (±0.75)	<0.0001
Energy (kcal/day)	2427.63 (±15.91)	2433.42 (±47.54)	2366.42 (±45.39)	<0.0001	1705.48 (±9.37)	1704.32 (±37.40)	1720.07 (±36.95)	<0.0001
Carbohydrate (g/day)	341.79 (±1.97)	341.99 (±6.79)	339.67 (±6.59)	<0.0001	267.51 (±1.50)	267.44 (±5.06)	268.39 (±4.95)	<0.0001
Protein (g/day)	87.98 (±0.78)	56.43 (±2.16)	53.92 (±2.07)	<0.0001	60.60 (±0.42)	38.82 (±1.50)	38.73 (±1.48)	<0.0001
Fat (g/day)	56.21 (±0.64)	88.46 (±2.10)	82.89 (±1.96)	<0.0001	38.82 (±0.37)	60.59 (±1.49)	60.67 (±1.47)	<0.0001
Dietary fiber (g/day)	27.04 (±0.21)	27.05 (±0.64)	26.99 (±0.62)	<0.0001	23.21 (±0.19)	23.29 (±0.59)	22.19 (±0.57)	<0.0001
Weight status				0.0014				<0.0001
Underweight	166 (2.4%)	153 (2.6%)	13 (1.3%)		430 (5.3%)	413 (5.6%)	17 (1.9%)	
Normal weight	2244 (29.9%)	2042 (30.2%)	202 (26.6%)		4158 (46.7%)	3963 (48.2%)	195 (27.6%)	
Overweight	1988 (26.0%)	1825 (26.3%)	163 (22.9%)		1982 (19.8%)	1844 (19.8%)	138 (19.3%)	
Obese	3036 (41.7%)	2711 (41.0%)	325 (49.1%)		2930 (28.3%)	2567 (26.4%)	363 (51.3%)	
Abdominal obesity status ^b^				<0.0001				<0.0001
No abdominal obesity	4908 (67.1%)	4507 (68.1%)	401 (56.7%)		6849 (75.3%)	6478 (77.0%)	371 (53.6%)	
Abdominal obesity	2526 (32.9%)	2224 (31.9%)	302 (43.3%)		2651 (24.7%)	2309 (23.0%)	342 (46.4%)	
Individual income level				0.0065				0.0814
Low	1769 (23.8%)	1556 (23.2%)	213 (30.0%)		2247 (23.9%)	2054 (23.6%)	193 (27.7%)	
Middle-lower	1838 (24.6%)	1664 (24.7%)	174 (23.6%)		2381 (24.6%)	2205 (24.6%)	176 (24.2%)	
Middle-upper	1902 (25.7%)	1745 (26.0%)	157 (22.6%)		2410 (25.0%)	2224 (25.0%)	186 (25.7%)	
High	1925 (25.9%)	1766 (26.1%)	159 (23.9%)		2462 (26.5%)	2304 (26.8%)	158 (22.5%)	
Education				<0.0001				0.0047
Elementary school	1110 (9.5%)	956 (9.0%)	154 (14.6%)		2344 (18.4%)	2115 (17.9%)	229 (23.9%)	
Middle school	771 (7.5%)	681 (7.3%)	90 (9.6%)		957 (8.8%)	890 (8.8%)	67 (8.3%)	
High school	2553 (37.3%)	2338 (37.6%)	215 (34.1%)		2969 (34.4%)	2764 (34.4%)	205 (34.2%)	
University or higher	3000 (45.7%)	2756 (46.1%)	244 (41.7%)		3230 (38.4%)	3018 (38.8%)	212 (33.6%)	
Marital status				0.0561				0.4859
Married	5927 (71.2%)	5341 (70.8%)	586 (75.3%)		8221 (80.8%)	7597 (80.7%)	624 (82.1%)	
Unmarried	1507 (28.8%)	1390 (29.2%)	117 (24.7%)		1279 (19.2%)	1190 (19.3%)	89 (17.9%)	
Drinking habits ^c^				0.0426				0.3976
None	1843 (24.6%)	1640 (24.1%)	203 (29.2%)		4023 (47.2%)	3719 (46.9%)	304 (50.4%)	
Moderate	2617 (39.7%)	2396 (40.0%)	221 (36.0%)		2884 (38.6%)	2685 (38.8%)	199 (36.2%)	
High	2636 (35.8%)	2396 (35.9%)	240 (34.8%)		1069 (14.2%)	990 (14.3%)	79 (13.4%)	
Smoking status				0.0601				0.1725
Never	1795 (26.5%)	1647 (26.9%)	148 (22.2%)		8533 (88.7%)	7910 (88.8%)	623 (86.5%)	
Past	3225 (38.4%)	2911 (38.4%)	314 (39.1%)		524 (5.9%)	480 (5.8%)	44 (6.2%)	
Current	2414 (35.0%)	2173 (34.7%)	241 (38.7%)		443 (5.5%)	379 (5.3%)	46 (7.3%)	
Total metabolic equivalent (MET min/week)	386.62 (±7.50)	392.58 (±23.94)	323.70 (±22.64)	<0.0001	362.68 (±6.32)	363.92 (±21.12)	347.24 (±20.24)	<0.0001
Aerobic physical activity status ^d^				0.0021				0.0975
Yes	3535 (51.8%)	3239 (52.4%)	296 (45.6%)		4002 (45.9%)	3728 (46.2%)	274 (42.3%)	
No	3899 (48.2%)	3492 (47.6%)	407 (54.4%)		5498 (54.1%)	5059 (53.8%)	439 (57.7%)	

Continuous variables are expressed as the mean with standard errors, and categorical variables are given as the number of subjects and percentages thereof. ^a^ The *p*-value was determined through categorical variables that were analyzed using chi-square tests, while continuous variables were assessed through *t*-tests. ^b^ The absence of abdominal obesity is indicated by a waist circumference below 90 cm for men or 85 cm for women, while the presence of abdominal obesity is identified by a waist circumference equal to or exceeding 90 cm for men or 85 cm for women. ^c^ Drinking habits: none; not drinking alcohol in the last year; and not drinking more than once a month; Moderate, drinking alcohol once a month or two to four times a month; High, drinking alcohol ≥2–3 times a week. ^d^ Aerobic exercise status: Yes, practice with an equivalent of 2 h 30 min of moderate-level exercise each week, or 1 h 15 min of high-level exercise, or mix high-level exercise with moderate-level exercise (1 min high-level exercise = 2 min moderate-level exercise). No, do not carry out all of the above activities.

**Table 2:** Characteristics of the study participants based on total physical activity ^a^.

	Men (n = 7434)					Women (n = 9500)				
	Inactive (n = 4370)	Somewhat Active (n = 922)	Active (n = 1440)	Very Active (n = 702)	*p*-Value ^b^	Inactive (n = 5408)	Somewhat Active (n = 1423)	Active (n = 1926)	Very Active (n = 743)	*p*-Value ^b^
Age (years)	47.8 (±0.67)	45.15 (±0.88)	43.18 (±0.79)	40.41 (±0.63)	<0.0001	49.66 (±0.70)	47.41 (±0.80)	45.13 (±0.76)	41.99 (±0.65)	<0.0001
Energy (kcal/day)	2424.84 (±51.16)	2398.91 (±58.99)	2393.15 (±54.76)	2538.21 (±46.92)	< 0.0001	1669.74 (±35.13)	1703.34 (±38.50)	1761.04 (±38.23)	1794.30 (±32.72)	<0.0001
Carbohydrate (g/day)	342.51 (±6.23)	336.96 (±7.21)	339.64 (±6.81)	347.72 (±5.65)	<0.0001	264.98 (±4.62)	268.54 (±5.36)	273.42 (±5.26)	267.08 (±4.30)	<0.0001
Protein (g/day)	86.14 (±3.68)	88.48 (±3.97)	86.89 (±3.69)	98.63 (±3.54)	<0.0001	58.52 (±1.74)	61.00 (±1.90)	63.01 (±1.83)	66.82 (±1.67)	<0.0001
Fat (g/day)	54.58 (±2.31)	54.72 (±2.50)	56.99 (±2.55)	64.63 (±2.19)	<0.0001	37.03 (±1.43)	38.58 (±1.58)	41.23 (±1.53)	44.30 (±1.33)	<0.0001
Dietary fiber	26.95 (±0.68)	27.24 (±0.79)	26.48 (±0.78)	28.32 (±0.62)	<0.0001	22.78 (±0.56)	23.49 (±0.64)	23.79 (±0.61)	23.95 (±0.53)	<0.0001
Ratio of macronutrients										
Carbohydrate (%)	63.69 (±0.22)	62.83 (±0.45)	62.49 (±0.38)	60.11 (±0.51)	<0.0001	66.05 (±0.21)	65.32 (±0.36)	64.45 (±0.29)	62.44 (±0.47)	<0.0001
Protein (%)	15.43 (±0.09)	15.92 (±0.20)	15.56 (±0.13)	16.50 (±0.24)	0.0001	14.39 (±0.08)	14.61 (±0.14)	14.69 (±0.11)	15.40 (±0.23)	<0.0001
Fat (%)	20.87 (±0.18)	21.26 (±0.37)	21.94 (±0.32)	23.39 (±0.40)	<0.0001	19.57 (±0.16)	20.07 (±0.29)	20.85 (±0.24)	22.16 (±0.39)	<0.0001
Weight status					0.2035					0.0921
Underweight	106 (2.6%)	25 (3.2%)	27 (2.1%)	8 (1.3%)		234 (5.3%)	64 (5.4%)	95 (5.5%)	37 (5.0%)	
Normal weight	1339 (30.1%)	277 (30.0%)	430 (30.0%)	198 (28.4%)		2285 (45.0%)	634 (47.0%)	881 (49.1%)	358 (50.6%)	
Overweight	1163 (25.8%)	240 (25.1%)	375 (25.7%)	210 (28.6%)		1136 (19.9%)	295 (19.7%)	395 (19.4%)	156 (19.9%)	
Obese	1762 (41.5%)	380 (41.7%)	608 (42.2%)	286 (41.7%)		1753 (29.8%)	430 (28.0%)	555 (26.1%)	192 (24.5%)	
Abdominal obesity status					0.0343					<0.0001
No abdominal obesity	2837 (65.9%)	607 (66.4%)	964 (68.7%)	500 (71.5%)		3746 (72.5%)	1044 (76.0%)	1457 (78.4%)	602 (83.5%)	
Abdominal obesity	1533 (34.1%)	315 (33.6%)	476 (31.3%)	202 (28.5%)		1662 (27.5%)	379 (24.0%)	469 (21.6%)	141 (16.5%)	
hs-CRP					0.027					0.8064
<3 mg/L	3911 (90.4%)	850 (92.4%)	1317 (92.2%)	653 (93.5%)		4988 (92.4%)	1320 (92.5%)	1783 (92.9%)	696 (93.4%)	
≥3 mg/L	459 (9.6%)	72 (7.6%)	123 (7.8%)	49 (6.5%)		420 (7.6%)	103 (7.5%)	143 (7.1%)	47 (6.6%)	

Continuous variables are expressed as the mean with standard errors, and categorical variables are given as the number of subjects and percentages thereof. ^a^ Based on the metabolic equivalents of tasks, participants were classified with total physical activity levels of 0–249 min per week as inactive, 250–499 min per week as somewhat active, 500–999 min per week as active, and ≥1000 min per week as very active. ^b^ *p*-value based on chi-square tests for categorical variables and a one-way ANOVA test for continuous variables.

**Table 3:** Odds ratios and 95% confidence intervals for high levels of hs-CRP (≥3 mg/L) according to dietary fiber intake and sedentary time.

	Model 1				
	Dietary Fiber (g/day)				
	Quartile 1	Quartile 2	Quartile 3	Quartile 4	*p*-Interaction
Men (n = 7434)					
Sedentary time (hours/day)					0.32
0–2	2.12 (0.91–4.98)	1.07 (0.42–2.68)	1.37 (0.54–3.46)	1.11 (0.45–2.73)	
3–6	0.90 (0.52–1.56)	0.65 (0.39–1.08)	0.81 (0.49–1.33)	1.09 (0.68–1.76)	
7–10	0.98 (0.59–1.63)	0.86 (0.54–1.37)	0.75 (0.46–1.21)	0.84 (0.53–1.33)	
11–22	Ref.	0.71 (0.42–1.20)	0.86 (0.50–1.47)	0.96 (0.58–1.61)	
Women (n = 9500)					
Sedentary time (hours/day)					0.41
0–3	1.20 (0.65–2.20)	0.24 (0.11–0.54)	0.82 (0.48–1.42)	0.77 (0.45–1.32)	
4–6	0.66 (0.41–1.09)	0.58 (0.35–0.97)	0.54 (0.34–0.87)	0.49 (0.30–0.79)	
7–10	0.76 (0.50–1.17)	0.80 (0.52–1.22)	0.66 (0.43–1.03)	0.67 (0.44–1.02)	
11–20	Ref.	0.61 (0.37–1.01)	0.73 (0.44–1.20)	0.57 (0.33–0.99)	
	**Model** **2**				
	**Dietary fiber (g/day)**				
	**Q** **uartile 1**	**Q** **uartile 2**	**Q** **uartile 3**	**Q** **uartile 4**	***p*-Interaction**
Men (n = 7434)					
Sedentary time (hours/day)					0.51
0–2	2.91 (0.97–8.67)	0.79 (0.29–2.20)	0.88 (0.34–2.33)	1.13 (0.42–3.03)	
3–6	0.99 (0.56–1.74)	0.57 (0.33–0.99)	0.79 (0.46–1.38)	1.04 (0.60–1.80)	
7–10	0.97 (0.57–1.66)	0.77 (0.47–1.27)	0.69 (0.41–1.17)	0.91 (0.54–1.55)	
11–22	Ref.	0.71 (0.41–1.25)	0.85 (0.48–1.50)	1.06 (0.58–1.95)	
Women (n = 9500)					
Sedentary time (hours/day)					0.09
0–3	0.81 (0.40–1.65)	0.24 (0.10–0.57)	0.67 (0.36–1.24)	0.51 (0.27–0.97)	
4–6	0.55 (0.32–0.95)	0.52 (0.29–0.91)	0.44 (0.26–0.75)	0.40 (0.22–0.73)	
7–10	0.69 (0.41–1.15)	0.78 (0.49–1.26)	0.62 (0.38–1.04)	0.53 (0.31–0.90)	
11–20	Ref.	0.62 (0.36–1.08)	0.58 (0.32–1.05)	0.32 (0.16–0.63)	

Model 1 reflects the unadjusted analysis. Model 2 is adjusted for age, income, education, drinking habits, smoking, marital status, aerobic exercise practice rate, and total daily energy intake.

**Table 4:** Odds ratios and 95% confidence intervals for high levels of hs-CRP (≥3 mg/L) according to dietary fiber intake and hand grip strength.

	Model 1					Model 2				
	Dietary Fiber (g/day)					Dietary Fiber (g/day)				
	Quartile 1	Quartile 2	Quartile 3	Quartile 4	*p*-Interaction	Quartile 1	Quartile 2	Quartile 3	Quartile 4	*p*-Interaction
Men (n = 7434)										
Average hand grip strength (kg)					0.38					0.48
Low (5.27–32.83)	Ref.	0.79 (0.51–1.22)	0.62 (0.39–0.99)	0.88 (0.55–1.42)		Ref.	0.81 (0.50–1.30)	0.64 (0.38–1.08)	0.96 (0.56–1.65)	
Middle (32.86–40)	0.66 (0.39–1.13)	0.46 (0.28–0.75)	0.56 (0.35–0.89)	0.59 (0.37–0.92)		0.78 (0.44–1.37)	0.49 (0.29–0.84)	0.65 (0.39–1.08)	0.67 (0.40–1.13)	
High (40.03–69.70)	0.61 (0.37–0.99)	0.34 (0.21–0.55)	0.44 (0.28–0.70)	0.61 (0.40–0.94)		0.75 (0.44–1.28)	0.41 (0.24–0.70)	0.53 (0.31–0.89)	0.77 (0.46–1.30)	
Women (n = 9500)										
Average hand grip strength (kg)					0.92					0.88
Low (5.27–19.43)	Ref.	0.67 (0.45–1.01)	0.75 (0.51–1.10)	0.64 (0.43–0.96)		Ref.	0.70 (0.44–1.12)	0.73 (0.47–1.14)	0.52 (0.31–0.86)	
Middle (19.46–23.96)	0.71 (0.45–1.11)	0.39 (0.25–0.60)	0.49 (0.33–0.74)	0.53 (0.36–0.79)		0.76 (0.45–1.29)	0.44 (0.27–0.71)	0.50 (0.31–0.82)	0.55 (0.34–0.90)	
High (23.97–44.70)	0.56 (0.36–0.87)	0.69 (0.46–1.04)	0.55 (0.37–0.82)	0.43 (0.28–0.64)		0.64 (0.39–1.04)	0.81 (0.51–1.28)	0.61 (0.38–0.97)	0.40 (0.24–0.68)	

Model 1 reflects the unadjusted analysis. Model 2 is adjusted for age, income, education, drinking habits, smoking, marital status, aerobic exercise practice rate, and total daily energy intake.

**Table 5:** Odds ratios and 95% confidence intervals for high levels of hs-CRP (≥3 mg/L) according to dietary fiber intake and resistance training.

	Model 1					Model 2				
	Dietary Fiber (g/day)					Dietary Fiber (g/day)				
	Quartile 1	Quartile 2	Quartile 3	Quartile 4	*p*-Interaction	Quartile 1	Quartile 2	Quartile 3	Quartile 4	*p*-Interaction
Men (n = 7434)										
Resistance training (times/week)					0.91					0.48
None	Ref.	0.80 (0.57–1.06)	0.74 (0.54–1.01)	0.98 (0.73–1.31)		Ref.	0.66 (0.47–0.92)	0.68 (0.48–0.96)	1.00 (0.68–1.47)	
1 to <3	0.42 (0.16–1.11)	0.30 (0.15–0.61)	0.40 (0.19–0.84)	0.75 (0.42–1.34)		0.41 (0.16–1.03)	0.31 (0.15–0.65)	0.35 (0.16–0.74)	0.78 (0.42–1.46)	
≥3	0.70 (0.39–1.26)	0.53 (0.32–0.89)	0.78 (0.50–1.22)	0.56 (0.37–0.85)		0.77 (0.42–1.42)	0.53 (0.30–0.92)	0.73 (0.43–1.24)	0.53 (0.32–0.89)	
Women (n = 9500)										
Resistance training (times/week)					0.32					0.51
None	Ref.	0.77 (0.58–1.04)	0.77 (0.59–1.02)	0.80 (0.62–1.05)		Ref.	0.86 (0.63–1.19)	0.77 (0.56–1.07)	0.67 (0.46–0.98)	
1 to <3	1.55 (0.79–3.04)	0.62 (0.27–1.46)	0.62 (0.32–1.20)	0.47 (0.19–1.16)		1.88 (0.93–3.80)	0.84 (0.35–2.02)	0.61 (0.29–1.27)	0.51 (0.19–1.32)	
≥3	0.55 (0.28–1.11)	0.70 (0.39–1.28)	0.94 (0.53–1.68)	0.40 (0.21–0.77)		0.64 (0.30–1.38)	0.73 (0.39–1.39)	1.01 (0.55–1.88)	0.40 (0.19–0.84)	

Model 1 reflects the unadjusted analysis. Model 2 is adjusted for age, income, education, drinking habits, smoking, marital status, aerobic exercise practice rate, and total daily energy intake.

**Table 6:** Odds ratios and 95% confidence intervals for high levels of hs-CRP (≥3 mg/L) according to dietary fiber intake and total physical activity levels.

	Model 1					Model 2				
	Dietary Fiber (g/day)					Dietary Fiber (g/day)				
	Quartile 1	Quartile 2	Quartile 3	Quartile 4	*p*-Interaction	Quartile 1	Quartile 2	Quartile 3	Quartile 4	*p*-Interaction
Men (n = 7434)										
Physical activity level ^a^					0.63					0.66
Inactive	Ref.	0.80 (0.58–1.12)	0.80 (0.57–1.12)	1.03 (0.75–1.41)		Ref.	0.69 (0.48–0.99)	0.69 (0.47–1.00)	0.99 (0.66–1.49)	
Somewhat active	0.52 (0.26–1.07)	0.78 (0.44–1.40)	0.64 (0.34–1.20)	0.78 (0.44–1.38)		0.54 (0.26–1.14)	0.63 (0.35–1.14)	0.69 (0.33–1.41)	0.69 (0.38–1.25)	
Active	1.01 (0.60–1.69)	0.50 (0.29–0.83)	0.71 (0.42–1.18)	0.75 (0.49–1.17)		1.00 (0.55–1.80)	0.46 (0.27–0.79)	0.62 (0.36–1.08)	0.84 (0.49–1.45)	
Very active	0.86 (0.41–1.79)	0.41 (0.16–1.04)	0.67 (0.36–1.24)	0.50 (0.27–0.95)		0.93 (0.43–2.00)	0.39 (0.15–1.06)	0.69 (0.36–1.30)	0.58 (0.28–1.18)	
Women (n = 9500)										
Physical activity level					0.18					0.15
Inactive	Ref.	0.90 (0.65–1.25)	0.73 (0.53–1.01)	0.66 (0.47–0.91)		Ref.	0.98 (0.69–1.41)	0.68 (0.46–1.00)	0.49 (0.32–0.75)	
Somewhat active	0.92 (0.53–1.60)	0.52 (0.29–0.91)	0.79 (0.46–1.34)	0.96 (0.59–1.58)		0.89 (0.46–1.63)	0.68 (0.38–1.23)	0.81 (0.45–1.43)	0.80 (0.44–1.46)	
Active	1.01 (0.63–1.62)	0.55 (0.33–0.93)	0.81 (0.54–1.21)	0.70 (0.46–1.07)		1.02 (0.58–1.78)	0.55 (0.31–0.96)	0.83 (0.50–1.37)	0.73 (0.43–1.24)	
Very active	0.87 (0.44–1.72)	0.55 (0.23–1.30)	0.83 (0.44–1.55)	0.58 (0.30–1.12)		1.04 (0.51–2.12)	0.54 (0.22–1.33)	0.86 (0.45–1.68)	0.51 (0.23–1.13)	

Model 1 reflects the unadjusted analysis. Model 2 is adjusted for age, income, education, drinking habits, smoking, marital status, and total daily energy intake. ^a^ Based on the metabolic equivalents of tasks, participants were classified with total physical activity levels of 0–249 min per week as inactive, 250–499 min per week as somewhat active, 500–999 min per week as active, and ≥1000 min per week as very active.

## Data Availability

The KNHANES dataset utilized in this study is publicly accessible at https://knhanes.kdca.go.kr/knhanes/eng/index.do (accessed on 20 August 2023).
